# Inpatient and emergency healthcare utilization and payments for care of SCD: an analysis of all payers in Florida 2010–2019

**DOI:** 10.1093/jscdis/yoaf039

**Published:** 2025-11-11

**Authors:** Linda Dynan, Richard B. Smith, Charles T. Quinn

**Affiliations:** 1 Department of Accounting, Economics and Finance, Haile College of Business Northern Kentucky University (NKU), Highland Heights, KY 41099, United States; 2 Anderson Center for Health Systems Excellence, Cincinnati Children’s Hospital Medical Center, Cincinnati, OH, 45229, United States; 3 Department of Economics, College of Arts & Sciences, University of South Florida, Tampa, FL, 33620, United States; 4 Division of Hematology, Cincinnati Children’s Hospital Medical Center, Cincinnati, OH, 45229, United States; 5 Department of Pediatrics, University of Cincinnati College of Medicine, Cincinnati, OH, 45229, United States

**Keywords:** SCD, health care utilization, insurance, socioeconomic status

## Abstract

Gene therapy offers transformative treatment for SCD, but access limitations necessitate continued reliance on traditional care, especially among pediatric and underserved populations. Thus, understanding healthcare utilization patterns and costs remains necessary for ongoing management and policy planning. We analyze all-payer emergency department (ED) and inpatient hospitalization data for Florida patients with SCD from 2010 to 2019. Payment estimates are constructed using novel charge-to-payment conversion methods. Outcomes are stratified by age (pediatric <12 years, adolescents 12–20 years, adults >20 years), insurance status, and income quartile. We find that ED visits for SCD increased by 95.7%, with the greatest growth among adults (106.3%). Mean ED payments rose across all age groups. Adult visits generated the highest average payments. Inpatient admissions grew by 13%, driven exclusively by adults. Mean inpatient length of stay (LOS) decreased across all age groups. SES analysis reveals persistent disparities, with the majority of ED visits and hospitalizations occurring in lower-income communities and among Medicaid (the payer with the lowest reimbursement rates) recipients. Our findings suggest that adults are increasingly the most resource-intensive users of healthcare, as successes in treatments have shifted the burden of care from children to adults. Shifts in health care resources have not appeared, however, to have kept pace with the changing demographics of the population with SCD (pediatric to adult care). ED use may have escalated particularly among adults because of the reduced access to prescription opioids for pain management in 2018 and lack of healthcare resources for non-pediatric SCD populations.

## INTRODUCTION

Transformative genetic treatments for SCD are now available for those 12 years of age and older. Initial prices listed at between 2.2 million and 3.1 million dollars for these products indicate traditional SCD therapies will continue for the foreseeable future. This is especially the case for those with SCD under age 12, and those who are ineligible or unwilling to undergo the new treatments.

This paper provides a new analysis of temporal trends in all-payer *payments* for traditional treatments in emergency department (ED) and inpatient hospitalization for the state of Florida, using a novel charge-to-payment conversion method to estimate the actual payments hospitals receive for these two modes of care. This is distinct from costs or charges. We describe differences between pediatric care (an ongoing concern as long as genetic treatment is unavailable before age 12), adolescent care (between 12 and 20), and adult care. We also evaluate the effects of socioeconomic status (SES) on payments for hospital care.

### Background and literature

There are an estimated 100 000 people with SCD in the US.^1^ More than 90% of this population is of African ancestry. Roughly 55% of patients with SCD are covered by Medicaid, and up to 40% of people with SCD qualify for publicly funded disability payments—and thus may have both Medicare and Medicaid (dual-eligible) insurance. Further, evidence suggests that Medicaid beneficiaries with SCD have higher healthcare utilization than those who are privately insured.^2^ Even within the Medicaid beneficiary population, the distribution of utilization and expenditures is uneven. The most severely ill, in the top 5% of expenditures (averaging 25 ED visits and 9 inpatient stays per year), approached $200 000 per patient in 2021.^3^

The National Opinion Research Center (NORC) at the University of Chicago^4^ analyzed 2021 Medicaid Claims (T-MSIS) data to assess SCD prevalence. Of the 52 547 Medicaid beneficiaries with SCD, the highest percentages of Medicaid enrollees with SCD were in Mississippi (0.20%), Georgia (0.17%), and Washington, DC (0.16%). However, almost 20% of all Medicaid beneficiaries with SCD were in just two states: New York (5033) and Florida (4877) (The Florida Agency for Health Care Administration (AHCA) estimated that number to be greater than 7000 in 2019).^5^

Advances in SCD treatment, including early introduction of hydroxyurea, now allow more than 95% of children born with SCD to reach adulthood (age 18).^6,7^ Recent published data estimate the average life expectancy among people with SCD to be 45 to 55 years,^8^ with the median age at death rising from 28 to 43 years. Deaths from SCD in younger age groups, although uncommon, are more likely to result from acute complications, while deaths from SCD are more common in adults and more likely to be related to chronic complications. This is consistent with the NORC^4^ reported age distribution of people with SCD covered by Medicaid, as approximately 51% of beneficiaries are pediatric (14% under 5; 18% between 6 and 12; and 19% between 13 and 20), with only about 10% over the age of 45.

Several studies have assessed costs and economic burden in SCD populations.^9–13^ However, data issues are complex and numerous. SCD populations are concentrated in several large and/or southern states. When small numbers of cases are used for sampling, statistically significant results may be identified that are not representative of the overall population because there is insufficient variation in the unweighted sample. These issues make it difficult to identify appropriate data sets and have influenced the choice of data sets (with limitations) to analyze. The existing literature, using a variety of available yet restricted data sets, has addressed ED and inpatient care utilization and costs, and to a lesser extent socioeconomic status. Table 1 briefly summarizes the literature with respect to data sources and major results of the papers (Table 1).

We report here a new analysis of ED and inpatient data for *all payers* in the state of Florida from 2010 to 2019. We construct *payment* estimates for ED and inpatient care in constant (ie, inflation-adjusted) 2019 dollars, using a *charge*-*to*-*payment* ratio of our own design. We focus on the overall use and payment for ED and inpatient care, describing trends in light of potential new treatments by stratifying our analyses by age per FDA gene therapy guidelines: pediatric (under twelve years old), adolescents (ages 12–20) and adults (over 20). We further examine the trends in pediatric, adolescent and adult populations of people with SCD with socioeconomic stratification based on (1) median-income quartile and (2) insurance status.

## METHODS

For this largely descriptive analysis, we use 2010 to 2019 all-payer hospital data. The dataset includes all ED visits and inpatient discharges, along with hospital financial information, from the Florida Agency for Healthcare Administration (AHCA). We use all ICD-9 and ICD-10 SCD codes as appropriate (excluding the code for sickle cell trait) to identify patients with SCD across all primary and secondary diagnoses. Because the data are hospital-level discharge data, they do not allow longitudinal tracking of individual patients. On the other hand, the availability of all ED and inpatient utilization data from this time period avoids the sampling issues (eg, weighting) identified above.

As a sensitivity test, we restrict the sample to only those discharges with a *primary* diagnosis of SCD.

We describe changes over time in payment and length of stay (LOS) for patients with SCD for both ED and inpatient use. We focus on payment for this all-payer dataset because costs do not vary as much by payer as payment does. We verify that charges (as a proxy for cost) do not vary substantially by payer in our data set, but payments do. We also assess the effects of socioeconomic status (SES), using an established method based on the median income of the patient’s ZIP code (see, for instance, the Agency for Healthcare Research and Quality [AHRQ] HCUP data).

### ED analysis methods

Our ED analysis includes all ED visits to Florida short-term general hospitals that did not lead to an inpatient admission at a facility in the same location.

We use individual-level ZIP codes to stratify patients by SES. Each observation links the patient’s ZIP code to the median income of that ZIP code between 2006 and 2010. These figures are based on the American Community Survey and provided by the Population Studies Center in the Institute for Social Research at the University of Michigan. We use these figures to present descriptive statistics by median-income quartile groups across all Florida ZIP codes.

We use hospital charges to estimate *what the hospital is actually paid for services*. While hospitals report charges, they almost never represent what is paid for the services provided. Payments are negotiated and generally vary by type of payer (eg, commercial insurance, Medicare, and Medicaid). Thus, we develop a hospital-based method of converting charges to payments based on the primary payer of the patient. The method is as follows, which applies elements of AHRQ’s Cost-to-Charge (to estimate hospital costs) formula for Emergency Department files:

1. Using AHCA financial data for each hospital and year, we calculate the portion of all outpatient revenues from each of the following major payers: Conventional Medicare, Medicare-HMO (ie, Medicare Managed Care), Conventional Medicaid, Medicaid-HMO, Other Government Insurance, Commercial Insurance, and Self Pay.
2. From the AHCA financial data, we obtain total ED revenues reported by each hospital for each year.
3. We multiply the portion of all outpatient revenues from each of the major payers (the ratio calculated in step 1) by total ED revenues to estimate annual ED revenues from each of the major payers for each hospital.
4. Using the AHCA ED discharge files, we sum total charges for each hospital by primary payer.
5. For each hospital, we divide annual ED revenues by total charges for each hospital by primary payer to obtain a charge-to-payment ratio (CPR) for each of the major payers.
6. For each ED discharge record, we multiply the appropriate CPR (based on the patient’s primary payer) by the reported charge in that record to estimate the visit payment (For a small percentage of discharges, the primary payer was not one of the major payers represented by our set of CPRs. In those cases, we used the most appropriate CPR; Kidcare (Florida’s CHIP program) is not separately identified as a payer for outpatient revenues. Therefore, the CPR for Conventional Medicaid is calculated as the conventional-Medicaid portion of ED revenues (which includes Kidcare revenues) divided by the sum of charges for conventional Medicaid and Kidcare. This same CPR is therefore applied to discharges for which the primary payer is either conventional Medicaid or Kidcare.).
7. Finally, we convert all monetary values to 2019 dollars using the consumer price index (CPI) from the Bureau of Labor Statistics.

Following AHRQ’s procedure for outliers in its cost-to-charge formula, we initially set to missing a CPR that was either negative or greater than 4. However, this would have caused masking approximately 12% of our total sample, and an even higher portion (15%) of our sample consisting of patients with SCD. Thus, rather than masking outlier CPRs, we replaced them with an overall CPR (total ED revenues divided by total charges). A test of the effect of replacing rather than masking outlier CPRs (and preserving 15% of our total sample) showed mean total payments changing by only 1%−2%.

Observations with inpatient admission from the ED are not included in the ED data. These are captured in the inpatient dataset.

### Inpatient analysis methods

The inpatient method is analogous, with appropriate modifications (Following AHRQ’s procedure for dealing with inpatient outliers in its Cost-to-Charge formula, we identify outlier records as those in which the payer-specific CPR is less than 0.05 or greater than 2. In those cases, we replace the payer-specific CPR with an overall CPR, which is total inpatient revenues divided by total inpatient charges.), to the ED method. The inpatient data capture all inpatient stays in Florida short-term general hospitals and include patient characteristics (age, percent Black, percent Medicaid [and Medicaid Managed Care], percent Medicare [and Medicare Managed Care], percent privately insured, and percent who self-pay or are uninsured); Admission Characteristics (percent admitted through the ED, and total LOS); Discharge Status (percent routinely discharged, percent discharged against medical advice [“own discharge”]), and percent who died during admission (ie, expired); and payments. We estimate payments received (using the CPR as described above) for each of 25 different categories of inpatient service, and use the same method identified in our ED analysis to present descriptive statistics by median-income quartile groups across all Florida ZIP codes.

To assess the accuracy of our estimates of both ED and inpatient payments, we conduct a secondary analysis, comparing our results to previously published estimates (see Appendix).

## RESULTS

### ED analysis

Our descriptive statistics for Florida indicate that among patients with SCD, ED use increased dramatically, by 95.7% for all ages, over the 2010–2019 period (Table 2). The pace of that increase rose with age: 43.3% under age 12; 95.9% ages 12–20; and 106.3% in the adult population. The large spike that occurs post 2018 coincides with the passage of Florida HB21 which limited the prescription of opioids for acute pain to a 3-day supply.^20^

Overall and within age groups, males increasingly account for ED visits over the study period. Within age categories, with the exception of adolescents, which is not statistically significant, the mean age of admission increased.

The share of ED claims paid by Medicaid overall in 2019 is about 50% with large variation by age group: almost 80% of claims for those under 12 years old have Medicaid as payer, compared to 44% of those 20 of age or older. The share of Medicaid as payer is statistically significant and increases for each age group from 2010 with the exception of pediatrics (under 12). This is paired with statistically significant declines in self-pay/charity care over the 2010 to 2019 period, again with the exception of pediatrics (under 12). Adolescents, who became eligible to remain on their parents’ insurance to age 26 under the Affordable Care Act (ACA) has the largest percentage decline in both self-pay and charity care associated with ED usage. Further reductions in self-pay and charity care may have been achieved if Florida had participated in the Medicaid expansions under the ACA.^21^

There is a statistically significant (*P* < .05) increase in mean length of stay (LOS) in the ED, rising overall by 19.3%, and rising in each age group. The largest increase in mean LOS is observed among pediatric patients, rising from 4.49 to 6.55 hours, an increase of 45.8%. Mean total payment likewise increases significantly over all age categories. Although the largest percentage increase in mean payment between 2010 and 2019 is in the pediatric group (81.1%) the highest mean payments are associated with the adult group ($2073). Fees for the three most expensive items—lab, radiology and ED—all increase, with the exception of radiology for the 12–20 age group, which falls by 6% ($10).

**Figure 1.**
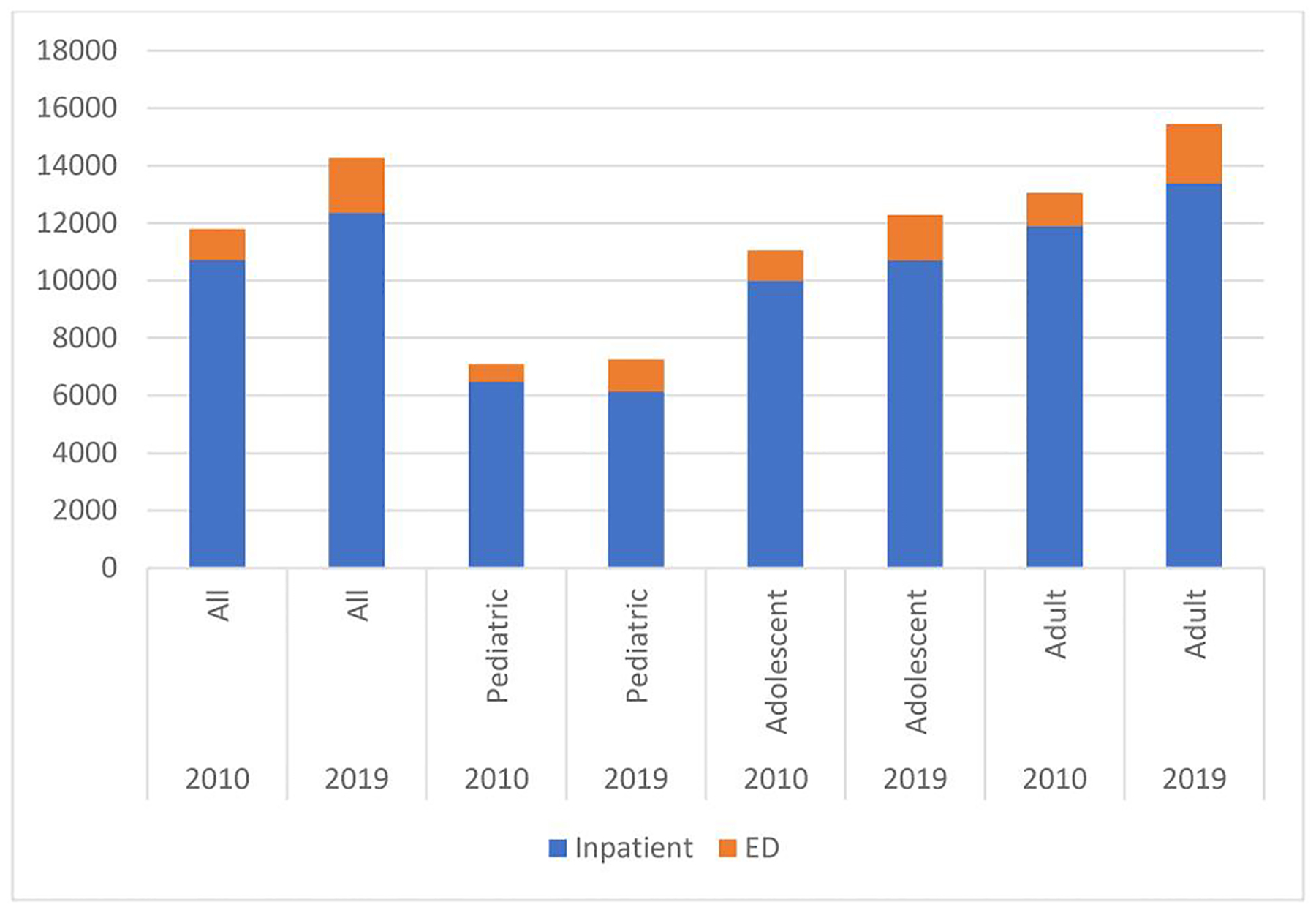
Mean total payments (2019 Dollars) by age group and year (2010 and 2019).

Payments in ED appear to be driven by an increase in average total payment per claim and by the large volume increases in each age category. However, the highest average total payment (level) is in the adult age group as is the largest percentage increase in volume (Figure 1), indicating a shift in the burden of SCD care from pediatrics to adults over time.

### Inpatient analysis

Overall, there is a 13% increase in inpatient admissions for patients with SCD between 2010 and 2019, but this is wholly in the adult population (see Table 3 for inpatient analysis.) Inpatient admissions for those under 20 *decrease* during the same period—for both pediatrics (34.3% decrease) and adolescents (14.4% decrease). In 2010, adults made up 71% of inpatient admissions. By 2019, adults account for 78% of inpatient admissions.

Similar to ED utilization, there is an increase in age within each category of inpatient admission and a decreased proportion of female inpatients.

Assessing inpatient admissions by insurance status, the share of payment by Medicaid decreases across all categories between 2010 and 2019, although non-significantly for adolescents. While self-pay and charity care percentages fall for ED visits, this is generally not true for inpatient admissions with the exception of charity care for adolescents (ages 12–20), potentially as a result of the ACA rule allowing children to stay on their parents’ insurance to age 26. The share of patients admitted to a hospital in the lowest median-income ZIP code quartile (the poorest group) also falls about 10% in each age group.

Inpatient admissions from the ED increase from 2010 to 2019: 54.9% overall, 62.8% for pediatrics, 49.3% for adolescents and 53.8% for adults. The overall proportion of admissions from the ED range from 86% to 90% by 2019. Mean hospital LOS decreases for all categories with the largest decline (18.8%) in adults. However, mean LOS is increasing with age, despite the overall decline from 2010 to 2019: 2.99 days in pediatrics, 4.1 days for adolescents and 4.67 days for adults in 2019.

Despite substantial decreases in mean LOS and pharmacy payments, down 23% overall, the mean total payment increases for all ages except pediatrics (a non-significant −5.1%). Mean total payments in 2019, like mean LOS, increases with age: $6,141 under 12; $10 708 for adolescents and $13 380 for adults (2.18 times that of pediatrics).

Between 2010 and 2019, the largest, and only, increase in inpatient admissions is for the most expensive group—adults. Even though the largest percentage increases in mean total payment is associated with ED use, inpatient total payments dwarfs those of the ED, and it is adults that lead the growth of inpatient total payments (Figure 1).

### SES and insurance status analysis

#### ED visits

**Figure 2.**
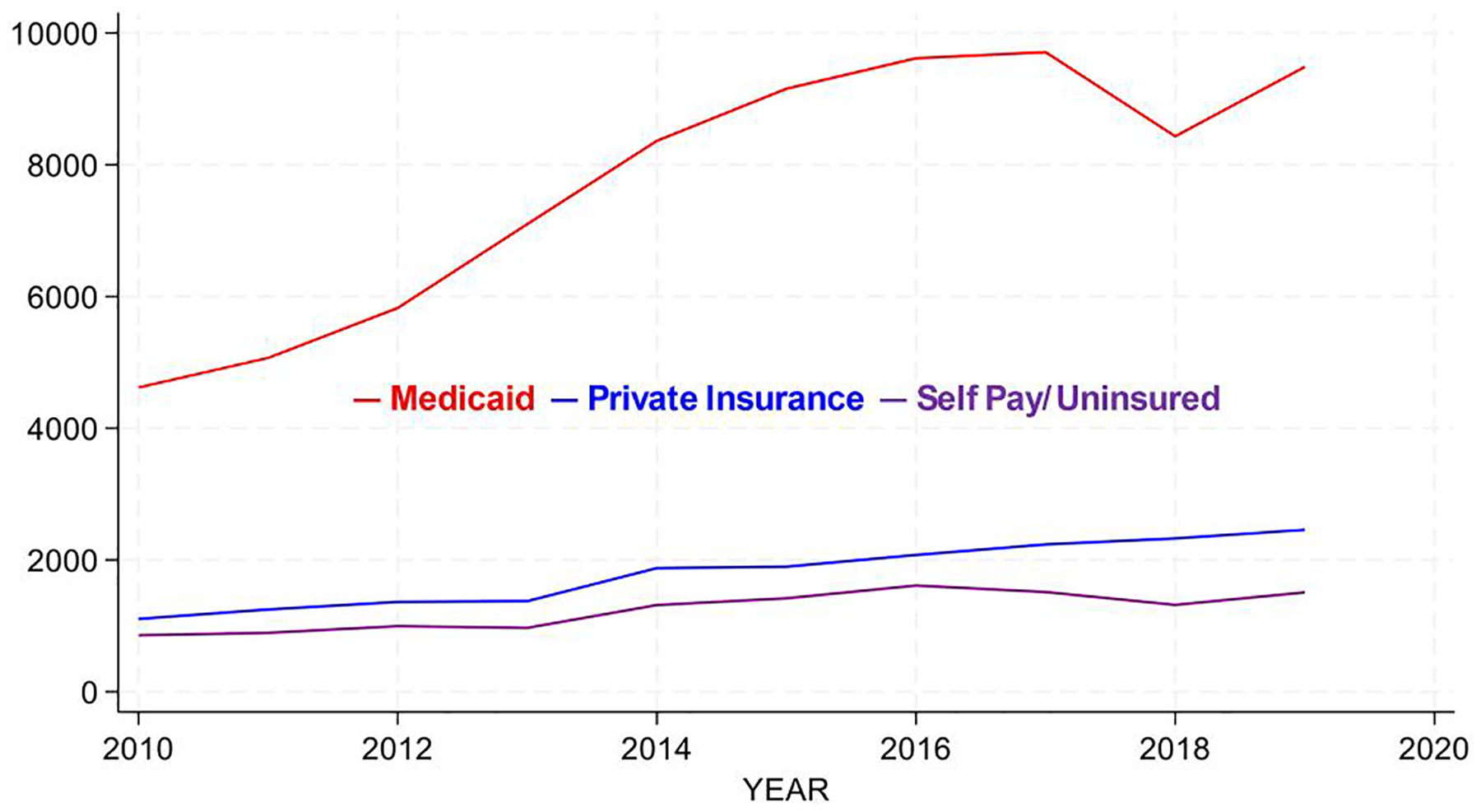
Total ED visits by insurance status for all ED patients (2010–2019).

Medicaid payer status dominates ED use across all age groups, and although it dips in 2018, it regains its upward trajectory in 2019 (Figure 2). Self-pay/uninsured admissions decline in 2017–2018. Analysis of median-income quartiles shows similar findings: the lowest income quartile dominates across all age groups for ED visits (graph not shown).

**Figure 3.**
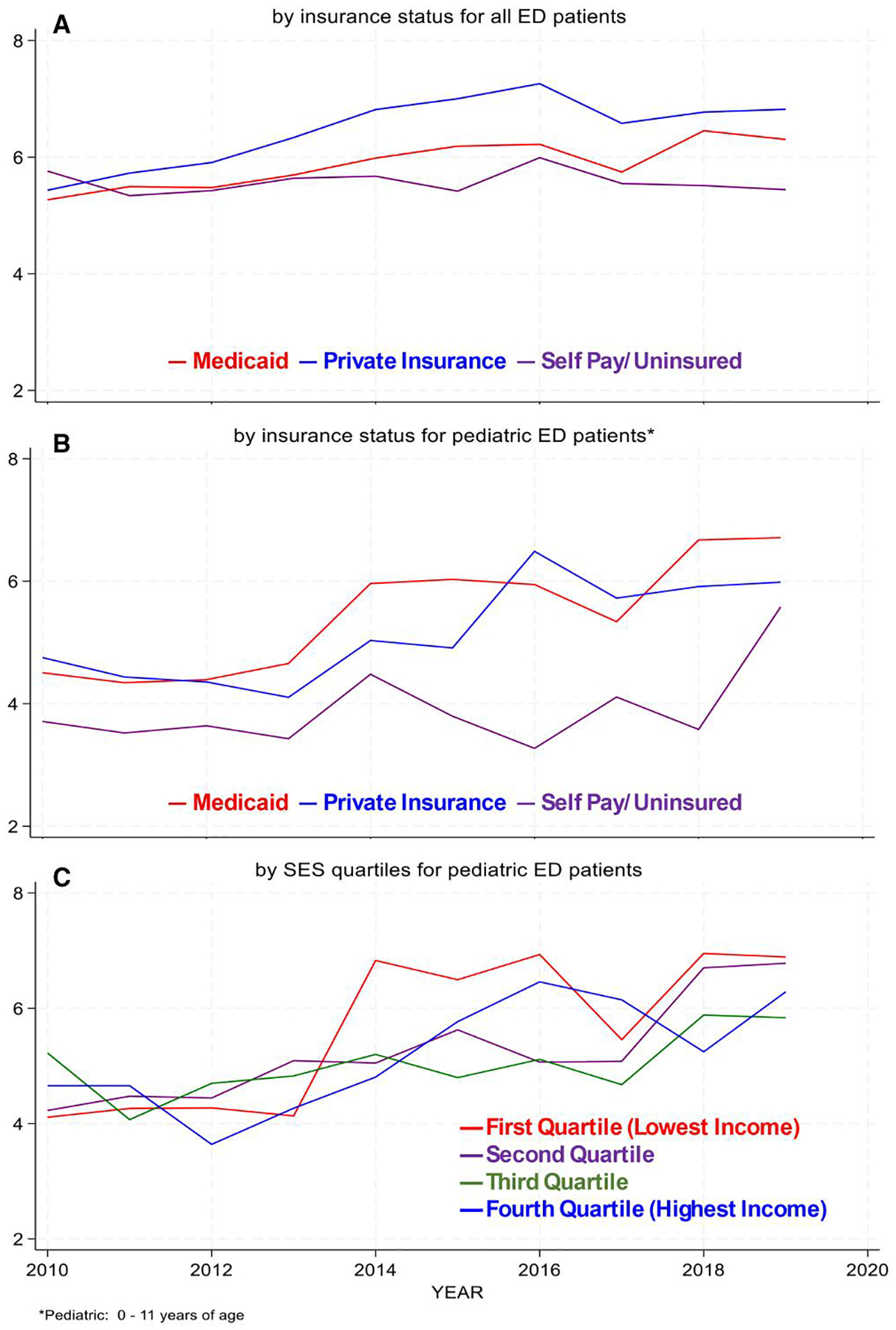
Length of stay (Hrs) for ED visits (2010–2019) Panel A: By insurance status for all ED patients; Panel B: By insurance status for pediatric ED patients; and Panel C: By SES quartile for pediatric ED patients.

ED LOS for adults and adolescents is roughly increasing for those privately insured and for those receiving Medicaid, with more volatility after 2016 (Figure 3A). Overall, ED LOS correlates with the level of reimbursement by payer (private, Medicaid). The LOS gap between privately insured and Medicaid narrows in the post-2016 periods. Analysis of LOS by median-income quartiles for adolescents and adults reinforces this—LOS is longest for the highest income quartile (figure not shown).

LOS appears linked to level of insurance payment more strongly in the adult and adolescent age groups. Noting that approximately 80% of pediatric ED visits are covered by Medicaid, we see the longest LOS associated with Medicaid for most of the 2010–2019 period with privately insured pediatric patients having longer LOS in 2011, and again between 2015 and 2017. However, LOS among the self-pay/uninsured (about 4%−5% of the pediatric ED visits) sharply rises towards the end of the period (Figure 3B).

Pediatric LOS by income quartile differs from the other two age groups, with the longest mean LOS associated with the two lowest income quartiles, although volatile over the period (Figure 3C).

#### Inpatient admissions

**Figure 4.**
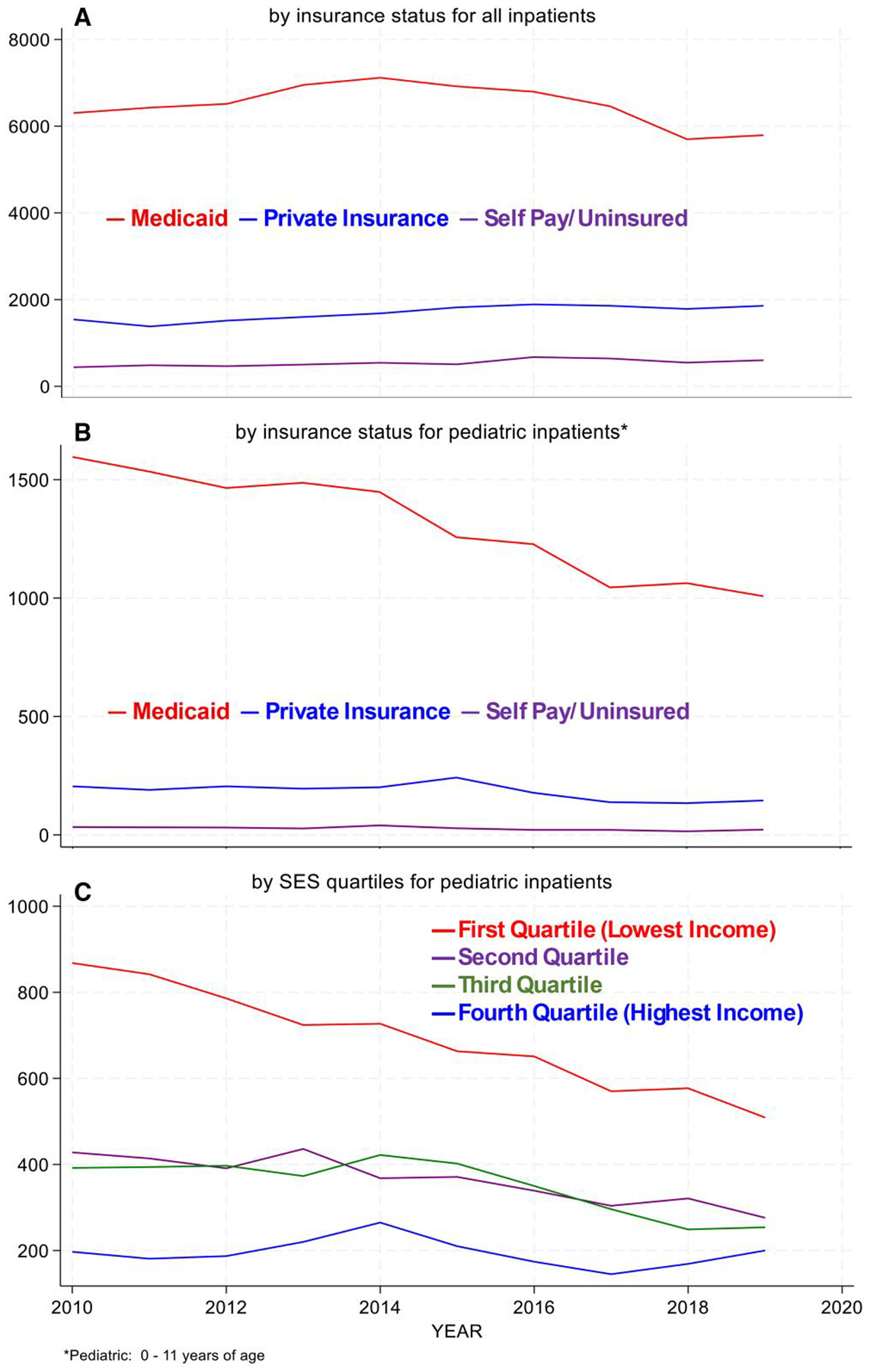
Inpatient Admissions (2010–2019) Panel A: By insurance status for all patients; Panel B: By insurance status for pediatric inpatients; and Panel C: By SES quartiles for pediatric inpatients.

Across patients of all ages, inpatient Medicaid admissions rise modestly until 2014, then decline to approximately 2010 levels by 2019, with relatively unchanging admissions for privately insured and self-pay/uninsured patients (Figure 4A). This is driven by the adult age group. Pediatric Medicaid inpatient admissions decline over the period (Figure 4B), which is reinforced for this age group when looked at by SES (Figure 4C).

#### Total payments

**Figure 5.**
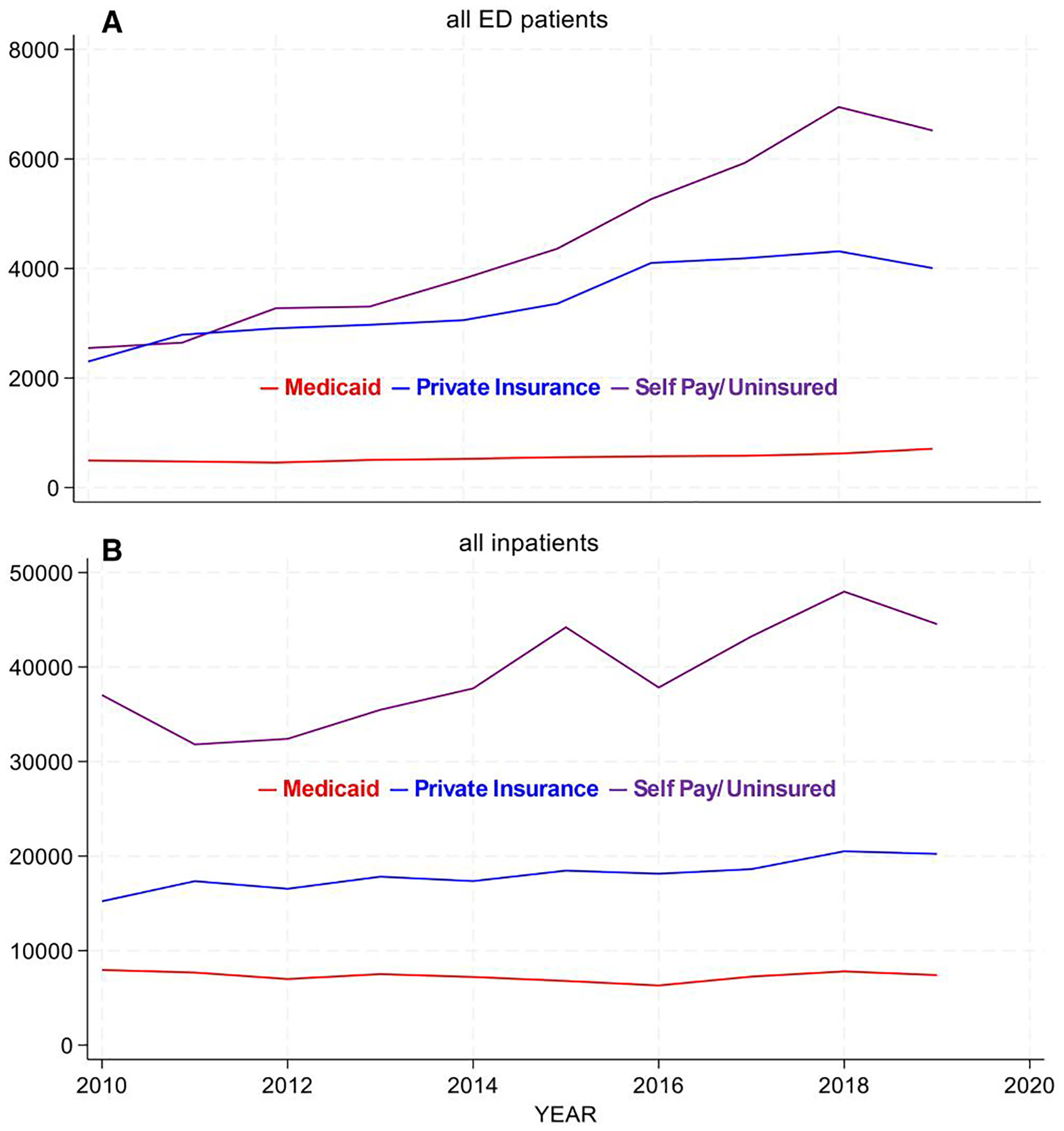
Total payments (2019 dollars) by insurance status (2010–2019) Panel A: All ED patients and Panel B All inpatients.

When evaluating the trend in total payments over the 2010–2019 period for ED visits, the most striking result is the low and static level of Medicaid payments (Figure 5A). By median-income quartiles (SES), higher income groups are associated with higher total payments, on a roughly increasing trajectory over time (graph not shown).

By payer for all inpatient stays (Figure 5B), total payments for the self-pay/uninsured follow an upward trajectory. Private payers have a much smaller upward trend relative to the self-pay/uninsured. Medicaid payments remain low and static, similar to ED payments. This pattern holds for all age groups.

Sensitivity analyses, where we restricted our sample of ED visits and inpatient discharges to those observations with a primary diagnosis of SCD (ICD9, ICD10) reveal similar patterns but with a 30%−40% drop in volume relative to the full sample.

## DISCUSSION

Although gene therapy is expected to eventually reduce the burden of lifetime care for persons with SCD, understanding of the trends in resource use and payments associated with traditional SCD care for both pediatric and adult populations is still highly relevant in a climate where gene therapy is not approved for those less than 12 years of age, and where eligible patients may face substantial barriers to access. This paper contributes to the SCD literature by focusing on resource use (ED visits and inpatient admissions) and *payments* (rather than costs or charges) by age group (pediatric, adolescent, and adult) and by socioeconomic status within age groups for all payers in Florida from 2010 to 2019. Payments across payers for the same services differ greatly, and such variability is not accounted for in studies of costs or charges.

We find that pediatric ED use and payments associated with SCD both increased in Florida over the 2010–2019 period, but the major determinant of growth in ED use was from the oldest age group (a 106.3% increase). Growth in the size of the adult population with SCD would be expected because as noted, current pediatric standard care now allows most patients with SCD to reach adulthood.^6,7^

However, payment by Medicaid is substantially lower than by private insurers or the self-pay/uninsured. In the pediatric case, almost 80% of claims for those under 12 years old have Medicaid as payer but only about 44% of those 20 years of age or older do, depressing payment to hospitals for the pediatric population.

Inpatient payments are substantially higher than ED payments for all age groups. Our findings, although somewhat higher, align with Adams-Graves and Bronte-Jordan,^6^ who find that adults aged 18–44 account for the majority (approximately two-thirds) of all SCD hospitalizations. We find a 24% increase in adult (over 20) inpatient admissions, while inpatient admissions for those under 20 *decreases* during the same period. Consequently, adults account for 71% of inpatient admissions at the start of the period (2010), and 78% in 2019.

Pediatric care has become an increasingly smaller share of both ED and inpatient use over time. The NORC data (focused on Medicaid beneficiaries) roughly indicate about 50% of the patient population is pediatric, but they account for only 11% of ED visits and 10% of inpatient admissions. Additionally, although adults’ total payments are higher on average than pediatric payments, a substantial proportion of adults with SCD are insured by Medicaid (about 44%), the lowest payer. Further, evidence suggests that Medicaid beneficiaries with SCD have higher healthcare utilization than those who are privately insured.^2^

The success of treatments allowing children with SCD to reach adulthood may have shifted care and payment burdens to later ages, with health care resources for SCD perhaps not keeping pace. Persons with SCD, particularly young adults, often present to the ED or hospital for SCD-related events as there is a paucity of primary care providers (PCP) who are willing or able to care for individuals with SCD.^17^ Lee et al^17^ report that access to specialized health care is limited for Medicaid recipients, citing in particular a retrospective claims analysis by Ballas et al^22^ that found that significantly fewer persons with Medicaid than with commercial insurance had visited a hematologist during the past year (7% with Medicaid vs 43% with commercial insurance *P* < .001).

The relatively large share of adults with SCD who are Medicaid beneficiaries (about 44% v 17.7% in 2019 for all Floridians^23^), in part because of the difficulties maintaining employment due to illness episodes, will be disproportionately impacted by the Act to Provide for Reconciliation pursuant to Title 11 of H. Con. Res. 14 (PL119–21) signed into law on July 4, 2025.^24^ Because Florida did not implement a Medicaid expansion under the ACA, the effects of PL119–21 will be less sharply felt than in expansion states such as New York, that has the largest number of Medicaid beneficiaries with SCD. This is because low-income Floridians were already deprived of the benefits associated with the original Medicaid expansion. However, PL 119–21 will have impacts for Floridians with SCD who have been insured through the ACA market places because PL 119–21institutes shorter enrollment periods, requires greater documentation to demonstrate eligibility, allows the enhanced premium credits to expire, and imposes monthly fees if beneficiaries automatically enroll without proactively verifying eligibility.

Although our study highlights a number of important findings, it does have limitations. In particular, we may have over-estimated sickle related acute care utilization because commonly used methods to validate SCD diagnoses by ICD coding^25^ cannot be used in this de-identified dataset. Our data are hospital-level discharge data that do not allow longitudinal tracking of individual patients, or the ability to capture nonhospital care. Further, our dataset is for the state of Florida. Our findings may be amplified or diminished in other states that experienced or implemented different policies than Florida (as noted for the ACA Medicaid expansions in New York).

## CONCLUSION

Our study focuses on the resource use and payments associated with SCD by age categories and SES. We find that the adult population is increasingly the most resource-intensive users of healthcare, suggesting that in recent years’ successes in treatments have extended life and have shifted the burden of care to from children to adults. The shift in health care resources for the population with SCD appears not to have kept pace however with the changing demographics.

Advocates for people with SCD should highlight the potential impacts of adverse changes in Medicaid policy recently enacted by Congress, given the substantial proportion of people with SCD reliant on Medicaid. Advocates should further highlight the changing demographics of persons with SCD and resources needed to meet these changes. Future work should examine how genetic therapies might influence overall resource use and payments for the entire SCD population.

## Supplementary Material

Supplemental Methods

Supplemental Figure 2

Supplemental Figure 1

Supplementary material is available at *Journal of Sickle Cell Disease* online.

## Figures and Tables

**Table 1.** Datasets used to evaluate SCD-related care utilization and costs.

Study	Data source	Data	Findings
Speller et al 2024^3^	Transformed Medicaid Statistical Information System (T-MSIS)	Medicaid enrollment, claims, and encounter data 2021	Average Medicaid spending for enrollees with SCD versus without SCD difference to be about two and a half times as much in a 2021 national sample
Peterson et al 2020^9^	Nationwide Inpatient Sample (NIS) and National Emergency Department Sample (NEDS)	2006 to 2015	Pediatric HbSS inpatient admissions through the ED had largest medical expenditure of SCD subtypes.
Kauf et al 2009^11^	Medicaid Claims data	Florida 2001–2005	80.5% of SCD-related healthcare costs were associated with inpatient hospitalizations
Johnson et al 2022^12^	Truven Health Marketscan and Medical Expenditure Panel Survey (MEPS)	A constructed retrospective cohort of commercial claims data from 2007 to 2018, matched controls from MEPS	Nonelderly lifetime burden of SCD medical costs: $1.7 million Non-elderly lifetime patient out of pocket costs: $44 000
Grady et al 2021^13^	MAX	CA 2014; FL 2012; NY 2013; and TX 2012	Average Medicaid spending for enrollees with SCD can be more than five times the average for all Medicaid enrollees in a state (e.g., $24 800 versus $4200 in Florida), ($22 600 versus $9175).
Shah et al 2019^14^	Medicaid Analytic eXtract (MAX)	All states 1/1/2009 to 12/31/2013	3+ VOEs are very costly
Gallagher et al 2022^15^	MarketScan Commercial, Medicare Supplemental, and Multi-state Medicaid Databases	1/1/2010 through 12/31/2018	Followed 4,487 patients (~79% Medicaid) for 5 years. Mean total healthcare costs were $275 143 (SD± $406 770) commercially insured and $362 728 (SD± $620 189) in the Medicaid sample; 18–30 year-olds were most expensive
Herring et al 2024^16^	Clinical Trial—Cost, quality-of-life, and other clinical data were sourced from HGB-206 data and the literature.	Patient-level simulation model using the phase 1/2 HGB-206 clinical trial (NCT02140554) as the basis for lovo-cel efficacy and safety.	Incremental cost-effectiveness ratios of patients treated with lovo-cel resulted in $191 519 and $124 051 per QALY gained from third-party payer and societal perspectives, respectively. However, despite meaningful improvements in survival, quality of life, and other clinical outcomes, overall costs for the health care system would increase.
Lee et al 2019^17^	Review Article		Unaddressed SCD complications and lack of a PCP associated with high rates of hospital readmission; a key barrier to successful transfer from pediatric to adult medical systems is the limited number of hematologists specializing in adults with SCD.
Ho et al 2019^18^	Nationwide Inpatient Sample (NIS)	2004–2012	Hospital admission rates and charges increased, hospital LOS decreased, while in-hospital mortality remained unchanged in the African-American population
McCavit et al 2011^19^	Nationwide Inpatient Sample (NIS)	2003–2005	Find hospital volume-outcomes effects for SCD inpatient treatment

**Table 2.** Descriptive statistics of ED visits for SCD in Florida (2010–2019).

							Descriptive Statistics, SCD Emergency Room Visits, 2010–2019																	
	All						Pediatric (<12 years of age)						Adolescent (12 to 20 years of age)						Adult (>20 years of age)					
	2010		2019		% Change		2010		2019		% Change		2010		2019		% Change		2010		2019		% Change	
	Mean	Std. Dev.	Mean	Std. Dev.		*P*	Mean	Std. Dev	Mean	Std. Dev		*P*	Mean	Std. Dev	Mean	Std. Dev		*P*	Mean	Std. Dev	Mean	Std. Dev.		*P*
**N (Number of Visits)**	9540		18 671				1419		2034				1117		2188				7004		14 449			
**Patient:**																								
Age	26.7	13.2	28.5	13.1	6.8%	.00***	4.8	3.4	5.0	3.4	3.7%	.12	17.3	2.6	17.1	2.6	−0.8%	.17	32.6	9.4	33.5	9.9	2.8%	.00***
Female	58%	49%	55%	50%	−5.3%	.00***	55%	50%	45%	50%	−16.9%	.00***	61%	49%	55%	50%	−9.6%	.00***	59%	49%	57%	50%	−3.4%	.01***
Black	95%	21%	94%	23%	−1.1%	.00***	91%	29%	90%	31%	−1.5%	.18	93%	25%	93%	26%	0.0%	.99	96%	19%	95%	22%	−1.5%	.00***
**Insurance Status:**																								
Medicaid	48.4%	50.0%	50.8%	50.0%	4.9%	.00***	80.7%	39.5%	79.6%	40.3%	−1.4%	.43	62.4%	48.5%	68.4%	46.5%	9.6%	.00***	39.6%	48.9%	44.1%	49.7%	11.2%	.00***
Medicare	28.0%	44.9%	24.9%	43.2%	−11.1%	.00***	0.0%	0.0%	0.4%	6.6%	0.0%	.01**	3.8%	19.2%	2.1%	14.5%	−44.2%	.00***	37.5%	48.4%	31.8%	46.6%	−15.3%	.00***
Private	11.6%	32.0%	13.2%	33.8%	13.7%	.00***	11.4%	31.8%	12.2%	32.7%	6.8%	.49	17.3%	37.8%	20.3%	40.2%	17.4%	.04**	10.7%	30.9%	12.2%	32.8%	14.1%	.00***
Other Gov. Insurance	1.6%	12.6%	2.3%	15.1%	43.7%	.00***	2.7%	16.1%	4.4%	20.5%	63.4%	.01***	2.8%	16.4%	2.8%	16.6%	2.1%	.92	1.2%	11.0%	2.0%	13.8%	60.8%	.00***
Self Pay (Uninsured)	9.0%	28.6%	8.1%	27.2%	−10.0%	.01***	4.9%	21.5%	3.1%	17.5%	−35.3%	.01***	12.0%	32.5%	5.8%	23.3%	−52.0%	.00***	9.3%	29.1%	9.1%	28.8%	−2.2%	.63
Nonpay (incl. Charity)	1.4%	11.9%	0.8%	8.7%	−46.7%	.00***	0.4%	5.9%	0.2%	5.0%	−30.2%	.57	1.7%	12.9%	0.6%	7.7%	−65.1%	.00***	1.6%	12.6%	0.9%	9.3%	−46.4%	.00***
**Socioeconomic Status (Zipcode Median-Income Quartile):**																								
First Quartile	45.0%	49.8%	46.1%	49.9%	2.4%	.08*	39.0%	48.8%	41.3%	49.3%	6.1%	.16	43.0%	49.5%	38.8%	48.7%	−9.8%	.02**	46.6%	49.9%	47.9%	50.0%	2.8%	.07*
Second Quartile	25.7%	43.7%	22.5%	41.8%	−12.5%	.00***	24.7%	43.2%	24.0%	42.7%	−3.0%	.62	23.7%	42.6%	27.4%	44.6%	15.6%	.02**	26.3%	44.0%	21.6%	41.1%	−17.9%	.00***
Third Quartile	22.3%	41.6%	22.7%	41.9%	1.8%	.44	26.8%	44.3%	23.8%	42.6%	−11.4%	.04**	21.5%	41.1%	22.5%	41.8%	4.9%	.49	21.5%	41.1%	22.6%	41.8%	4.9%	.08*
Fourth Quartile	6.9%	25.4%	8.6%	28.1%	25.0%	.00***	9.4%	29.3%	10.9%	31.1%	15.1%	.18	11.8%	32.3%	11.3%	31.7%	−4.5%	.65	5.6%	23.0%	7.9%	27.0%	41.0%	.00***
**Visit Characteristics:**																								
Primary Diagnosis of SCD	67.16%	46.97%	70.02%	45.82%	4.3%	.00***	47.43%	49.95%	50.98%	50.00%	7.5%	.04**	59.18%	49.17%	68.46%	46.48%	15.7%	.00***	72.43%	44.69%	72.94%	44.43%	0.7%	.43
SCD Center Affiliation	20.6%	40.4%	21.3%	40.9%	3.5%	.16	31.9%	46.6%	34.4%	47.5%	8.0%	.12	23.5%	42.4%	26.5%	44.1%	12.6%	.07*	17.8%	38.2%	18.6%	38.9%	4.8%	.13
Hematologist in Patient Zipcode	37.7%	48.5%	42.8%	49.5%	13.4%	.00***	37.6%	48.5%	45.2%	49.8%	20.1%	.00***	40.0%	49.0%	39.5%	48.9%	−1.3%	.77	37.4%	48.4%	42.9%	49.5%	14.8%	.00***
Weekend (Saturday or Sunday)	28%	45%	26%	44%	−5.0%	.01**	26%	44%	27%	44%	3.4%	.56	25%	43%	24%	43%	−1.4%	.83	29%	45%	27%	44%	−7.0%	.00***
Blood Transfusion	8.2%	27.4%	8.6%	28.0%	4.3%	.31	19.7%	39.8%	23.8%	42.6%	20.6%	.01***	8.8%	28.3%	8.8%	28.4%	0.5%	.96	5.8%	23.3%	6.4%	24.4%	10.4%	.09*
Routine Discharge	93.4%	24.8%	90.4%	29.4%	−3.2%	.00***	92.0%	27.1%	92.9%	25.7%	0.9%	.36	92.3%	26.7%	92.0%	27.2%	−0.4%	.73	93.9%	23.9%	89.9%	30.2%	−4.3%	.00***
Transfer to Inpatient	1.6%	12.6%	1.9%	13.7%	18.1%	.08*	5.4%	22.7%	4.5%	20.7%	−17.6%	.20	3.5%	18.4%	3.6%	18.5%	2.1%	.91	0.5%	7.3%	1.3%	11.3%	138.5%	.00***
Own Discharge	4.1%	19.9%	6.5%	24.7%	58.2%	.00***	0.8%	9.2%	0.8%	8.8%	−7.0%	.85	2.6%	15.9%	2.3%	14.9%	−12.0%	.58	5.0%	21.8%	8.0%	27.1%	58.5%	.00***
Patient Expired	0.063%	2.5%	0.043%	2.1%	−31.9%	.47	0.211%	4.6%	0.049%	2.2%	−76.7%	.17	0.000%	0.0%	0.000%	0.0%	#DIV/0!	1.00	0.043%	2.1%	0.048%	2.2%	13.1%	.86
Length of Stay (Hours)	5.37	5.97	6.41	9.43	19.3%	.00***	4.49	5.21	6.55	10.53	45.8%	.00***	5.24	5.65	6.66	10.01	27.1%	.00***	5.57	6.14	6.35	9.17	14.0%	.00***
Total Payment	$1082	$1699	$1912	$4207	76.7%	.00***	$618	$815	$1120	$2908	81.1%	.00***	$1057	$2192	$1584	$3463	49.9%	.00***	$1180	$1726	$2073	$4442	75.7%	.00***
**Sub-Categories of Total Payment**																								
Pharmacy	$87	$163	$112	$383	29.2%	.00***	$66	$130	$51	$173	−22.5%	.01***	$90	$216	$111	$506	23.3%	.19	$91	$159	$121	$383	33.6%	.00***
Medical Supply	$23	$95	$12	$178	−46.9%	.00***	$12	$56	$3	$45	−72.0%	.00***	$21	$178	$4	$45	−80.5%	.00***	$25	$81	$14	$201	−42.4%	.00***
Laboratory	$248	$430	$392	$879	58.2%	.00***	$189	$306	$323	$763	70.6%	.00***	$268	$521	$375	$890	39.9%	.00***	$257	$434	$405	$892	57.7%	.00***
Radiology	$156	$687	$291	$1604	87.0%	.00***	$76	$199	$110	$459	44.7%	.01***	$189	$895	$178	$739	−6.0%	.70	$167	$711	$334	$1790	100.5%	.00***
ER	$443	$630	$761	$1372	71.7%	.00***	$229	$323	$439	$957	91.9%	.00***	$386	$618	$646	$1392	67.5%	.00***	$496	$668	$824	$1411	66.2%	.00***
OR	$2	$70	$16	$986	789.4%	.15	$2	$72	$11	$374	467.1%	.38	$5	$149	$1	$22	−84.6%	.19	$1	$46	$19	$1111	1395.5%	.17
Anesthesia	$1	$29	$2	$88	240.4%	.06*	$1	$37	$2	$79	133.2%	.53	$2	$58	$0	$5	−92.8%	.16	$0	$17	$3	$96	499.6%	.04**
Trauma	$3	$114	$4	$217	7.3%	.92	$3	$58	$2	$83	−36.0%	.68	$7	$242	$0	$0	−100.0%	.16	$3	$87	$4	$245	56.9%	.60
Recovery	$0	$11	$2	$50	435.7%	.01**	$0	$7	$1	$41	641.0%	.30	$1	$25	$0	$6	−81.9%	.15	$0	$8	$2	$55	756.6%	.01**
Cardiology	$5	$169	$8	$199	54.9%	.22	$0	$11	$0	$0	−100.0%	.03**	$3	$69	$2	$49	−24.2%	.75	$7	$195	$10	$225	54.0%	.25
Gastro Intestinal	$0	$23	$1	$43	50.5%	.71	$1	$53	$0	$5	−91.6%	.28	$0	$0	$0	$0	0.0%	1.00	$0	$13	$1	$48	266.3%	.43
Lithotripsy	$0	$14	$1	$37	90.6%	.43	$0	$10	$0	$0	−100.0%	.10*	$0	$4	$1	$39	563.3%	.55	$0	$15	$1	$39	93.7%	.48
Treatment-Observation	$11	$114	$79	$721	621.0%	.00***	$10	$104	$107	$973	1018.5%	.00***	$9	$94	$102	$1216	1047.1%	.01**	$12	$118	$71	$561	518.5%	.00***
Other	$103	$283	$232	$661	125.4%	.00***	$29	$126	$71	$306	142.1%	.00***	$77	$203	$165	$466	115.6%	.00***	$122	$313	$265	$716	116.9%	.00***

*** *P*<.01.

** *P*<.05.

* *P*<.1.

**Table 3.** Descriptive statistics of inpatient admissions for SCD in Florida (2010–2019).

Descriptive statistics, SCD inpatient admissions, 2010–2019																								
	All						Pediatric (<12 years of age)						Adolescent (12 to 20 years of age)						Adult (>20 years of age)					
	2010		2019		% Change		2010		2019		% Change		2010		2019		% Change		2010		2019		% Change	
	Mean	Std. Dev.	Mean	Std. Dev.		*P*	Mean	Std. Dev.	Mean	Std. Dev.		*P*	Mean	Std. Dev.	Mean	Std. Dev.		*P*	Mean	Std. Dev.	Mean	Std. Dev.		*P*
**N (Number of Admissions)**	11 384		12 860				1885		1239				1886		1613				8063		10 008			
**Patient**																								
Age	26.5	14.6	30.9	15.7	16.8%	.00***	4.7	3.5	5.4	3.6	13.8%	.00***	17.0	2.5	17.1	2.5	0.9%	.08*	33.8	11.2	36.3	13.2	7.5%	.00***
Female	58%	49%	56%	50%	−2.3%	.03**	47%	50%	43%	49%	−9.0%	.02**	59%	49%	54%	50%	−7.9%	.01***	60%	49%	58%	49%	−2.6%	.03**
Black	93%	26%	93%	26%	0.3%	.39	85%	36%	94%	24%	10.4%	.00***	93%	26%	92%	26%	−0.3%	.74	94%	23%	93%	26%	−1.6%	.00***
**Insurance Status**																								
Medicaid	53.3%	49.9%	45.0%	49.8%	−15.4%	.00***	84.7%	36.0%	81.4%	39.0%	−3.9%	.02**	74.8%	43.4%	74.7%	43.5%	−0.1%	.97	40.9%	49.2%	35.8%	47.9%	−12.5%	.00***
Medicare	26.4%	44.1%	32.0%	46.6%	20.8%	.00***	0.0%	0.0%	0.2%	4.9%	0.0%	.03**	1.6%	12.7%	1.7%	13.1%	5.6%	.83	38.4%	48.6%	40.7%	49.1%	6.1%	.00***
Private	13.0%	33.7%	14.4%	35.2%	10.8%	.00***	10.9%	31.1%	11.7%	32.2%	7.6%	.47	15.3%	36.0%	15.6%	36.3%	2.3%	.77	13.0%	33.6%	14.6%	35.3%	12.1%	.00***
Other Gov. Insurance	1.9%	13.6%	2.6%	15.8%	35.6%	.00***	2.1%	14.2%	3.7%	18.9%	79.4%	.01***	3.6%	18.5%	3.7%	18.9%	4.7%	.79	1.5%	12.0%	2.2%	14.8%	52.9%	.00***
Self Pay (Uninsured)	3.7%	18.9%	4.7%	21.1%	25.9%	.00***	1.8%	13.1%	1.8%	13.2%	1.4%	.96	3.1%	17.4%	3.3%	17.8%	5.0%	.79	4.3%	20.3%	5.3%	22.3%	22.0%	.00***
Nonpay (incl. Charity)	1.7%	12.8%	1.3%	11.4%	−20.2%	.03**	0.6%	8.0%	1.2%	10.9%	90.2%	.09*	1.6%	12.7%	0.9%	9.6%	−43.4%	.07*	1.9%	13.6%	1.4%	11.7%	−26.3%	.01***
**Socioeconomic Status (Zipcode Median-Income Quartile)**																								
First Quartile	46.0%	49.8%	41.5%	49.3%	−9.9%	.00***	46.0%	49.9%	41.1%	49.2%	−10.8%	.01***	42.4%	49.4%	37.4%	48.4%	−11.9%	.00***	46.9%	49.9%	42.2%	49.4%	−10.0%	.00***
Second Quartile	22.9%	42.0%	24.6%	43.1%	7.3%	.00***	22.7%	41.9%	22.3%	41.6%	−1.9%	.78	23.8%	42.6%	28.4%	45.1%	19.5%	.00***	22.8%	41.9%	24.3%	42.9%	6.5%	.02**
Third Quartile	20.7%	40.5%	22.6%	41.8%	9.2%	.00***	20.8%	40.6%	20.5%	40.4%	−1.4%	.84	20.0%	40.0%	23.4%	42.3%	16.9%	.02**	20.8%	40.6%	22.7%	41.9%	9.2%	.00***
Fourth Quartile	10.4%	30.5%	11.4%	31.8%	9.5%	.01**	10.5%	30.6%	16.1%	36.8%	54.5%	.00***	13.8%	34.5%	10.8%	31.1%	−21.6%	.01***	9.6%	29.4%	10.9%	31.1%	13.5%	.00***
**Admission Characteristics**																								
Primary Diagnosis of SCD	72.78%	44.51%	70.25%	45.72%	−3.5%	.00***	61.59%	48.65%	59.48%	49.11%	−3.4%	.24	79.22%	40.59%	79.48%	40.40%	0.3%	.85	73.89%	43.92%	70.09%	45.79%	−5.1%	.00***
SCD Center Affiliation	23.53%	42.42%	21.59%	41.15%	−8.2%	.00***	27.16%	44.49%	21.47%	41.08%	−21.0%	.00***	29.11%	45.44%	25.29%	43.48%	−13.1%	.01**	21.38%	41.00%	21.01%	40.74%	−1.7%	.55
Hematologist in Patient Zipcode	40.63%	49.12%	40.37%	49.06%	−0.6%	.67	42.49%	49.45%	50.52%	50.02%	18.9%	.00***	40.40%	49.08%	45.51%	49.81%	12.6%	.00***	40.25%	49.04%	38.28%	48.61%	−4.9%	.01***
ER Admit	57.77%	49.40%	89.49%	30.67%	54.9%	.00***	52.84%	49.93%	86.04%	34.67%	62.8%	.00***	59.01%	49.19%	88.10%	32.39%	49.3%	.00***	58.63%	49.25%	90.14%	29.82%	53.8%	.00***
Experienced Preventable Complication (PSI 3, 6, 7, 8, 9, 10, 11, 12, 13, 14, or 15)	0.32%	5.66%	0.04%	1.97%	−87.9%	.00***	0.00%	0.00%	0.00%	0.00%	0.0%	1.00	0.27%	5.14%	0.00%	0.00%	−100.0%	.04**	0.41%	6.38%	0.05%	2.23%	−87.8%	.00***
Routine Discharge	90.28%	29.62%	85.17%	35.54%	−5.7%	.00***	98.36%	12.72%	98.06%	13.79%	−0.3%	.54	94.64%	22.52%	93.74%	24.23%	−1.0%	.25	87.37%	33.22%	82.19%	38.26%	−5.9%	.00***
Transfer to Inpatient	0.64%	7.99%	0.95%	9.69%	47.7%	.01***	1.01%	9.99%	0.97%	9.80%	−3.9%	.91	1.01%	9.99%	1.30%	11.34%	29.2%	.41	0.47%	6.85%	0.89%	9.39%	88.7%	.00***
Own Discharge	3.77%	19.04%	6.49%	24.64%	72.3%	.00***	0.00%	0.00%	0.16%	4.02%	0.0%	.08*	1.86%	13.50%	2.23%	14.78%	20.3%	.43	5.10%	22.00%	7.96%	27.07%	56.2%	.00***
Patient Expired	0.44%	6.61%	0.58%	7.56%	31.0%	.13	0.11%	3.26%	0.00%	0.00%	−100.0%	.25	0.32%	5.63%	0.19%	4.31%	−41.5%	.44	0.55%	7.37%	0.71%	8.39%	30.0%	.17
Length of Stay (Days)	5.19	4.26	4.43	3.73	−14.6%	.00***	3.31	2.74	2.99	2.28	−9.6%	.00***	4.72	3.99	4.10	3.28	−13.1%	.00***	5.74	4.47	4.67	3.89	−18.8%	.00***
Total Payment	$10 715	$16 911	$12 347	$22 115	15.2%	.00***	$6471	$13 951	$6141	$8702	−5.1%	.46	$9985	$20 013	$10 708	$21 597	7.2%	.30	$11 878	$16 581	$13 380	$23 188	12.6%	.00***
**Sub-Categories of Total Payment**																								
Room and Board	$1697	$2291	$1824	$3397	7.5%	.00***	$1191	$1544	$1213	$1665	1.8%	.71	$1758	$2252	$1907	$3688	8.5%	.14	$1801	$2426	$1887	$3499	4.8%	.06*
Pharmacy	$2854	$6353	$2198	$6362	−23.0%	.00***	$1325	$4359	$793	$1652	−40.2%	.00***	$2696	$8024	$2177	$6618	−19.3%	.04**	$3248	$6248	$2375	$6659	−26.9%	.00***
Medical Supply	$497	$1609	$355	$2347	−28.6%	.00***	$267	$818	$196	$2072	−26.5%	.18	$437	$1258	$168	$782	−61.6%	.00***	$565	$1804	$405	$2537	−28.4%	.00***
Laboratory	$1955	$3192	$2355	$4380	20.4%	.00***	$1244	$2769	$1310	$1862	5.3%	.46	$1767	$3670	$2067	$4871	17.0%	.04**	$2166	$3138	$2530	$4497	16.8%	.00***
Radiology	$958	$2196	$1326	$4040	38.4%	.00***	$459	$1445	$371	$1025	−19.1%	.06*	$834	$2323	$1043	$3792	25.0%	.05**	$1104	$2291	$1490	$4286	35.0%	.00***
ER	$569	$653	$873	$1464	53.6%	.00***	$366	$422	$503	$771	37.4%	.00***	$522	$617	$752	$1424	44.2%	.00***	$627	$695	$938	$1526	49.7%	.00***
OR	$288	$1406	$462	$3544	60.4%	.00***	$165	$1022	$209	$1365	27.1%	.30	$236	$1272	$190	$1634	−19.3%	.36	$329	$1507	$537	$3931	63.1%	.00***
Anesthesia	$76	$410	$121	$803	59.5%	.00***	$56	$361	$71	$374	26.4%	.27	$78	$401	$56	$382	−27.9%	.10	$80	$422	$137	$887	72.4%	.00***
Trauma	$2	$69	$3	$103	64.9%	.35	$0	$0	$3	$63	0.0%	.08*	$3	$102	$9	$219	180.2%	.32	$2	$67	$2	$74	3.3%	.96
Recovery	$60	$360	$76	$501	27.3%	.00***	$38	$224	$43	$218	15.1%	.49	$62	$347	$35	$234	−43.7%	.01***	$64	$388	$86	$555	35.0%	.00***
Cardiology	$132	$894	$221	$1346	67.5%	.00***	$29	$184	$62	$870	115.6%	.11	$79	$369	$125	$802	58.5%	.03**	$169	$1062	$256	$1458	52.0%	.00***
Nursery I	$2	$155	$0	$13	−92.4%	.30	$10	$388	$1	$42	−87.3%	.45	$0	$0	$0	$0	0.0%	1.00	$0	$2	$0	$0	−100.0%	.12
Nursery II	$6	$222	$4	$300	−37.1%	.54	$35	$556	$36	$965	5.0%	.95	$0	$0	$0	$0	0.0%	1.00	$0	$7	$0	$0	−100.0%	.27
Nursery III	$0	$24	$2	$125	361.5%	.25	$2	$61	$18	$402	663.0%	.10*	$0	$0	$0	$0	0.0%	1.00	$0	$0	$0	$0	0.0%	1.00
ICU	$499	$2055	$940	$3610	88.3%	.00***	$326	$1634	$280	$1335	−14.3%	.40	$381	$1748	$665	$2919	74.3%	.00***	$567	$2201	$1066	$3882	88.0%	.00***
Coronary	$130	$1125	$294	$1679	126.6%	.00***	$38	$803	$266	$898	596.3%	.00***	$109	$1722	$344	$1482	216.7%	.00***	$156	$1005	$289	$1780	85.3%	.00***
Oncology	$1	$22	$0	$0	−100.0%	.00***	$0	$0	$0	$0	0.0%	1.00	$0	$12	$0	$0	−100.0%	.23	$1	$26	$0	$0	−100.0%	.00***
Respiratory Therapy	$298	$1722	$254	$1290	−14.8%	.02**	$637	$2515	$360	$1232	−43.5%	.00***	$379	$1781	$297	$1572	−21.7%	.15	$200	$1446	$234	$1244	17.0%	.09*
Physical Therapy	$24	$174	$43	$286	78.7%	.00***	$6	$67	$6	$52	−2.1%	.96	$23	$151	$33	$261	44.8%	.15	$29	$195	$50	$306	71.6%	.00***
Occupational Therapy	$8	$140	$20	$225	152.6%	.00***	$4	$61	$11	$69	176.0%	.00***	$9	$115	$17	$187	100.1%	.09*	$9	$158	$22	$243	148.8%	.00***
Speech Therapy	$2	$39	$10	$141	393.1%	.00***	$3	$47	$1	$30	−57.5%	.26	$3	$57	$4	$111	48.7%	.65	$2	$32	$11	$153	648.4%	.00***
Labor	$24	$378	$30	$540	26.4%	.29	$0	$0	$0	$0	0.0%	1.00	$19	$202	$30	$489	56.5%	.38	$31	$447	$34	$579	11.1%	.67
Treatment-Observation	$58	$328	$157	$682	169.6%	.00***	$18	$140	$51	$282	179.4%	.00***	$57	$320	$137	$920	139.2%	.00***	$68	$359	$174	$671	155.6%	.00***
Behavioral Health	$1	$17	$1	$16	−6.5%	.86	$0	$1	$1	$27	3967.0%	.09*	$1	$19	$1	$20	33.6%	.71	$1	$19	$0	$13	−41.2%	.27
Other	$576	$1448	$768	$1711	33.4%	.00***	$253	$780	$335	$712	32.5%	.00***	$532	$1278	$642	$1444	20.8%	.02**	$662	$1588	$842	$1825	27.2%	.00***

*** *P*<.01.

** *P*<.05.

* *P*<.1.
