# Supplementary material for: Inpatient and emergency healthcare utilization and payments for care of SCD: an analysis of all payers in Florida 2010–2019
Source: J Sick Cell Dis. Author manuscript; Available in PMC 2025 Dec 13. (PMC12700639; doi:10.1093/jscdis/yoaf039)
Supplement: Supplemental Methods [file NIHMS2128011-supplement-Supplemental_Methods.docx]

**Appendix: Analysis of Hospital Payments**

**Inpatient and emergency healthcare utilization and payments for care of sickle cell disease: an analysis of all payers in Florida 2010-2019**

**Linda Dynan, PhD**

Department of Accounting, Economics and Finance, Haile College of Business Northern Kentucky University (NKU), 330 BAC Highland Heights, Kentucky, 41099 USA

Anderson Center for Health Systems Excellence, Cincinnati Children's Hospital Medical Center, Cincinnati, Ohio, USA

[dynanl@nku.edu](mailto:dynanl@nku.edu)

**Richard B Smith, PhD**

Department of Economics, College of Arts & Sciences, University of South Florida, Tampa, Florida, USA

**Charles T Quinn, MD, MS**

Division of Hematology, Cincinnati Children's Hospital Medical Center, Cincinnati, OH, USA.

Department of Pediatrics, University of Cincinnati College of Medicine, Cincinnati, OH, USA.

To assess the accuracy of our estimate of hospital payments, we compared our results to those of recent studies producing similar estimates. Selden (2020) used responses from the Medical Expenditure Panel Survey (MEPS), between 2010 and 2016, to report mean actual payments for inpatient admissions and emergency department (ED) visits, by payer source.^1^ In our supplementary analysis, we compare his results to corresponding estimates from our data using all Florida hospital ED visits (approximately 35 million visits) and inpatient admissions (approximately 15 million admissions) over the same time period. Because the Selden figures are based on a nationally representative survey sample, we calculate 95 confidence intervals for his payments using standard errors reported by the author.

For emergency department (ED) services, mean Medicaid payments in Florida and the U.S. (Selden) are relatively constant between 2010 and 2016, with our estimate of payments in Florida about half to two-thirds the U.S. average (Figure S1-a.). This is consistent with Florida being among the five lowest states in terms of Medicaid payments per beneficiary).^2^ For inpatient services, there is effectively no difference, with Florida Medicaid payments within the 95-percent confidence interval of U.S. Medicaid payments throughout the time period (Figure S1-b). In contrast, our estimate of payments for private insurance are substantially higher than the U.S. sample reported by Selden, for both ED and inpatient services.

We also looked, therefore, at a study by Bai and Anderson (2018), who used the same Florida hospital financial data as in this study (from AHCA) to construct a *relative price*, specifically, the ratio of private to Medicare revenues, by hospital and year, between 2010 and 2016.^3^ The authors found private revenues to increase from 2.0 to 2.6 times Medicare revenues, for all private short-term hospitals, between 2010 and 2016. To compare, we constructed a similar measure based on our estimate of mean annual private-insurance and Medicare payments for all ED and inpatient services, across all public and private short-term hospitals. We find that private payments increased from about 1.6 to 2.2 times Medicare payments, for both ED and inpatient services, between 2010 and 2016 (Figure S2, panels a. and b.). While our estimates are not directly comparable to Bai and Anderson due to differences in measurement and the population of hospitals studied (Bai and Anderson studied only private hospitals), both sets of analyses find a similar rate of increase in relative price (private-to-Medicare payments) between 2010 and 2016.

Based on this review of, and comparison to, previously published estimates, we believe our method of converting hospital charges to payments is sound and reasonably accurate.

**References**

Selden, T. M. Differences between public and private hospital payment rates narrowed, 2012-

16. *Health Aff (Millwood)* 39, no. 1 (Jan 2020): 94-99.

<https://doi.org/10.1377/hlthaff.2019.00415>.<https://www.ncbi.nlm.nih.gov/pubmed/31905058>.

2Kaiser Family Foundation. KFF analysis of the T-MSIS Research Identifiable Files, 2021,

[https://www.kff.org/medicaid/state-indicator/medicaid-spending-by-enrollment group/?currentTimeframe=0&sortModel=%7B%22colId%22:%22Location%22,%22sort%22:%22asc%22%7D](https://www.kff.org/medicaid/state-indicator/medicaid-spending-by-enrollment%20group/?currentTimeframe=0&sortModel=%7B%22colId%22:%22Location%22,%22sort%22:%22asc%22%7D).

3Bai, G., and G. F. Anderson. Market power: Price variation among commercial insurers for

hospital services. *Health Aff (Millwood)* 37, no. 10 (Oct 2018): 1615-22.

<https://doi.org/10.1377/hlthaff.2018.0567>. <https://www.ncbi.nlm.nih.gov/pubmed/30273037>.

**Figure Legends**

Figure S1. Medicaid and Private Hospital Payments (2010-2016)

Figure S2. Private-Insurance to Medicare Payments (2010-2016)
