# Supplementary figures and images for: Inpatient and emergency healthcare utilization and payments for care of SCD: an analysis of all payers in Florida 2010–2019
Source: J Sick Cell Dis. Author manuscript; Available in PMC 2025 Dec 13. (PMC12700639; doi:10.1093/jscdis/yoaf039)

Figure S2. Private-Insurance to Medicare Payments (2010-2016)

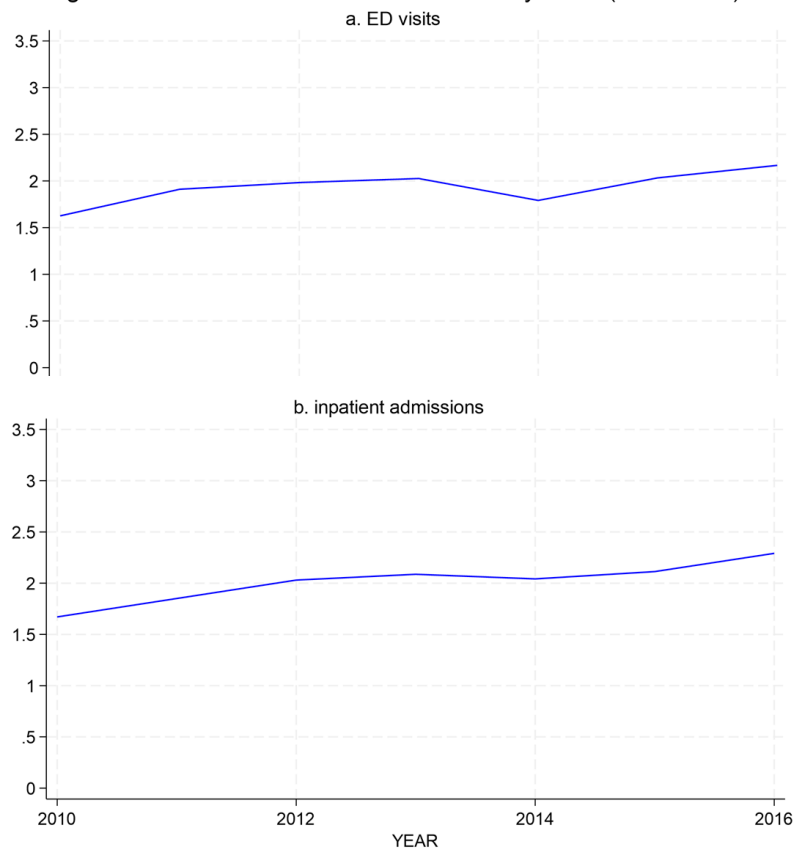

Supplement: Supplemental Figure 2 [file NIHMS2128011-supplement-Supplemental_Figure_2.pdf]

Figure S1. Medicaid and Private Hospital Payments (2010-2016)

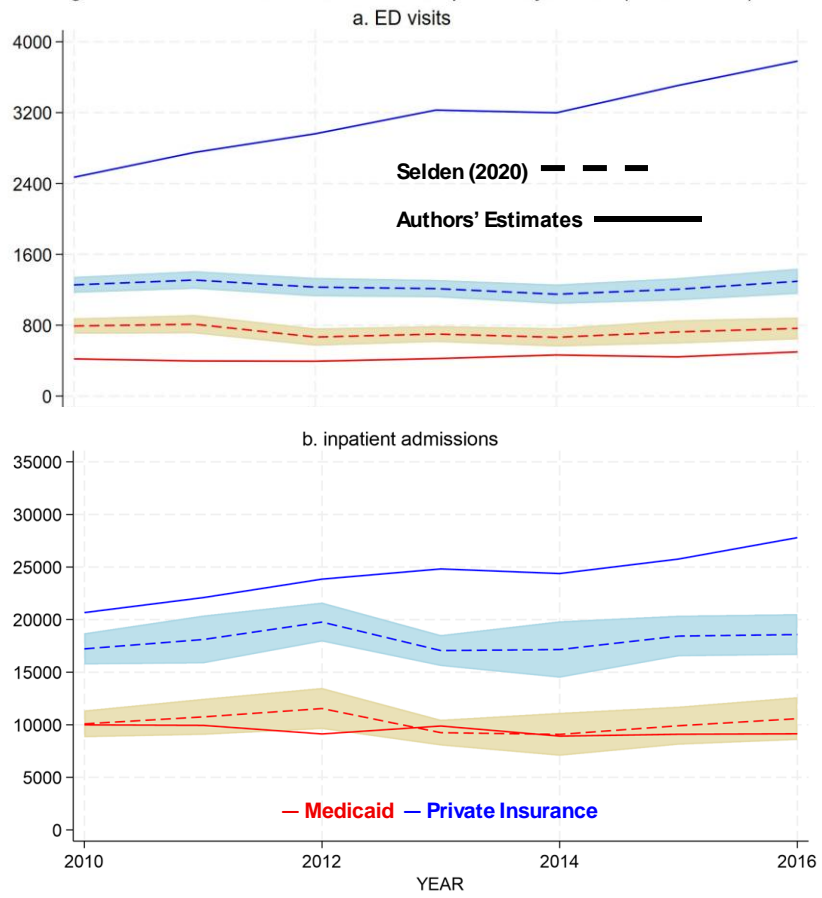

Supplement: Supplemental Figure 1 [file NIHMS2128011-supplement-Supplemental_Figure_1.pdf]
